# The c.429_452 duplication of the *ARX* gene: a unique developmental-model of limb kinetic apraxia

**DOI:** 10.1186/1750-1172-9-25

**Published:** 2014-02-14

**Authors:** Aurore Curie, Tatjana Nazir, Amandine Brun, Yves Paulignan, Anne Reboul, Karine Delange, Anne Cheylus, Sophie Bertrand, Fanny Rochefort, Gérald Bussy, Stéphanie Marignier, Didier Lacombe, Catherine Chiron, Mireille Cossée, Bruno Leheup, Christophe Philippe, Vincent Laugel, Anne De Saint Martin, Silvia Sacco, Karine Poirier, Thierry Bienvenu, Isabelle Souville, Brigitte Gilbert-Dussardier, Eric Bieth, Didier Kauffmann, Philippe Briot, Bénédicte de Fréminville, Fabienne Prieur, Michel Till, Caroline Rooryck-Thambo, Isabelle Mortemousque, Isabelle Bobillier-Chaumont, Annick Toutain, Renaud Touraine, Damien Sanlaville, Jamel Chelly, Sonya Freeman, Jian Kong, Nouchine Hadjikhani, Randy L Gollub, Alice Roy, Vincent des Portes

**Affiliations:** 1 Centre de Référence « Déficiences Intellectuelles de Causes Rares », Hôpital Femme Mère Enfant, Hospices Civils de Lyon, F-69677 Bron, France; 2 CNRS UMR 5304, L2C2, Institut des Sciences Cognitives, F- 69675 Bron, France; 3 Université de Lyon, Faculté de médecine Lyon Sud - Charles Mérieux, F-69008 Lyon, France; 4 Athinoula A Martinos Center for Biomedial Imaging, Massachusetts General Hospital, Charlestown, MA, USA; 5 Génétique, Bordeaux, France; 6 INSERM UMR 663, Hôpital Necker-Enfants malades, Paris, France; 7 Génétique moléculaire, Strasbourg, France; 8 Génétique, Nancy, France; 9 Pédiatrie, Strasbourg, France; 10 Neuropédiatrie, Hôpital Trousseau, Paris, France; 11 Génétique moléculaire, Hôpital Cochin, Paris, France; 12 Génétique, Poitiers, France; 13 Génétique, Toulouse, France; 14 Fondation Sonnenhof, Bischwiller, France; 15 MAS de Courcouronnes, Courcouronnes, France; 16 Génétique, Saint-Etienne, France; 17 Médecine interne, Hôpital Saint Luc Saint Joseph, Lyon, France; 18 Génétique, Tours, France; 19 Service de génétique, Hôpital Femme Mère Enfant, Hospices Civils de Lyon, CRNL, CNRS UMR5292, INSERM U1028, Université Claude Bernard Lyon I, Lyon, France

**Keywords:** *ARX* gene mutation, Kinematic study, Limb-kinetic apraxia, X-linked intellectual disability, Partington syndrome

## Abstract

**Background:**

The c.429_452dup24 of the *ARX* gene is a rare genetic anomaly, leading to X-Linked Intellectual Disability without brain malformation. While in certain cases c.429_452dup24 has been associated with specific clinical patterns such as Partington syndrome, the consequence of this mutation has been also often classified as “non-specific Intellectual Disability”. The present work aims at a more precise description of the clinical features linked to the c.429_452dup24 mutation.

**Methods:**

We clinically reviewed all affected patients identified in France over a five-year period, i.e. 27 patients from 12 different families. Detailed cognitive, behavioural, and motor evaluation, as well as standardized videotaped assessments of oro-lingual and gestural praxis, were performed. In a sub-group of 13 *ARX* patients, kinematic and MRI studies were further accomplished to better characterize the motor impairment prevalent in the *ARX* patients group. To ensure that data were specific to the *ARX* gene mutation and did not result from low-cognitive functioning per se, a group of 27 age- and IQ-matched Down syndrome patients served as control.

**Results:**

Neuropsychological and motor assessment indicated that the c.429_452dup24 mutation constitutes a recognizable clinical syndrome: *ARX* patients exhibiting Intellectual Disability, without primary motor impairment, but with a very specific upper limb distal motor apraxia associated with a pathognomonic hand-grip. Patients affected with the so-called Partington syndrome, which involves major hand dystonia and orolingual apraxia, exhibit the most severe symptoms of the disorder. The particular “reach and grip” impairment which was observed in all *ARX* patients, but not in Down syndrome patients, was further characterized by the kinematic data: (i) loss of preference for the index finger when gripping an object, (ii) major impairment of fourth finger deftness, and (iii) a lack of pronation movements. This lack of distal movement coordination exhibited by *ARX* patients is associated with the loss of independent digital dexterity and is similar to the distortion of individual finger movements and posture observed in Limb Kinetic Apraxia.

**Conclusion:**

These findings suggest that the *ARX* c.429_452dup24 mutation may be a developmental model for Limb Kinetic Apraxia.

## Background

The *Aristaless Related homeoboX (ARX)* gene is one of the most important genes responsible for X-Linked Intellectual Disability (XLID) [1-4]. Depending on the type of mutation, *ARX* gene-related loss of function leads to a pleiotropy. Nonsense mutations, for instance, are responsible for severe brain malformations (due to total loss of GABAergic neurons) and severe convulsive encephalopathies such as XLAG syndrome (X-linked Lissencephaly with Abnormal Genitalia; OMIM 300215) [5-10]. Expansion of the first repeated PolyA sequence of the *ARX* gene by insertion of 7 GCG triplets (c.333ins(GCG)_7_) leads to early infantile epileptic encephalopathy with suppression burst (Ohtahara syndrome, OMIM 308350) [11,12], or infantile epileptic-dyskinetic encephalopathy without obvious brain malformation [13-16].

The most prevalent *ARX* mutation is a duplication of 24 bp in exon 2 (c.429_452dup24), which leads to an increase from 12 to 20 alanines in the second polyalanine tract in the ARX protein [17]. This duplication occurs in 67%-76% of all unrelated *ARX* mutated patients without brain malformation [18,19], considering the potential for ascertainment bias as the gene sequencing is not routinely available. This mutation can be associated with either West syndrome (OMIM 308350) [2,13,20] or Partington syndrome (OMIM 309510). The latter combines ID with a very particular “dystonia” that affects fingers and wrist movements, but has no effect on the movements of the trunk and lower limbs [21]. Patients with Partington syndrome also have impaired oro-lingual praxis, leading in the most severe forms to anarthria and permanent salivary drooling [21,22]. However, most patients with the c.429_452dup24 mutation have been classified as having “non specific XLID” (OMIM 300419) because accompanying motor impairment was not obvious [1,3,18,19,23-29]. After clinical re-evaluation, several case reports revealed though that patients previously described as non specific did in fact present mild “dystonic” features as well [22,24,30,31].

To better characterize the c.429_452dup24 *ARX* mutation consequences, we assessed all affected patients identified in France over a five-year period (27 patients from 12 different families) using standardized cognitive and behavioural tests and an extensive evaluation of motor skills, including videotaped clinical evaluation of oro-lingual and gestural praxis. In response to the particular “reach and grasp” impairment, which seemed to be a pathognomonic sign in this population, we performed a kinematic and MRI study in a sub-group of 13 patients, in order to better characterize the phenotype. We also included a group of 27 age- and IQ-matched Down syndrome patients who served as controls to ensure our data were not resulting from low-cognitive functioning, but were specific to the *ARX* gene mutation.

## Patients and methods

### Patients

*Recruitment procedure:* Families and patients were notified of the study by the physician who ordered the genetic analysis. After we informed the patients and their parents or guardians about the aims of the study, all of them gave written informed consent in accordance with the Ethical Committee protocols of French Public Hospitals (CPP DGS2007-0131, 04/18/2007) before the study procedure started.

#### Patients with the c.429_452dup24 ARX mutation later designated as ‘ARX patients’

*Molecular screening:* Mutation analysis on genomic DNA was done as previously described [3]. During the five-year inclusion period (2002–2006), 13 families were diagnosed with the c.429_452dup24 mutation in the *ARX* gene in France. Twelve families (Additional file 1: Figure S1) agreed to be revisited. Five of these families (two sporadic cases, three brother pairs) had not been studied previously. The remaining seven families had participated in other studies [1,3,31]. Only one family (two brothers) declined to participate in the study. Twenty-seven males were included in the clinical study (age range: 19 months to 56 years). All 27 patients were examined by the same child neurologist (AC) either at home or at the nearest hospital.

#### Down syndrome patients (DS)

To test the specificity of the fine motor skill impairment and cognitive profile observed in the *ARX* patients, we included a control group of 27 age- and IQ-matched Down syndrome patients who did not have other severe medical conditions (uncontrolled seizures or unstable heart defect).

#### Healthy controls

A group of 13 age-matched healthy control subjects with no history of neurological or psychiatric disease was recruited to perform the kinematic task and undergo the morphometric MRI.

### Clinical assessment

***Exhaustive clinical data*** were collected from the caregiver as well as the patient’s medical records, including: pedigree, birth parameters, early development, fine motor skills, language, school curriculum, behavioural troubles, antipsychotic and anticonvulsant medications, seizure types and frequency.

***Clinical examinations*** included dysmorphological and neurological assessments, especially fine motor skills and orolingual praxis.

***Cognitive, adaptive, and behavioral skills*** were assessed in *ARX* and age-matched DS patients.

### Motor assessment

#### Videotaped oro-lingual and gestural praxis protocol

A gestural and orolingual praxis scale was specifically developed for the present study (Additional file 2: Table S1). The scale includes fifteen items, five focused on orolingual praxis and ten focused on gestural praxis. Given that a difference of one point regarding the mean of the oro-lingual and gestural score was meaningful and a statistical significance criterion of 0.05, we computed that in order to get a power of 95% the sample size of the population included in the study should be greater than 17. We decided to include 20% more. Twenty-one *ARX* and 21 age- and IQ-matched DS patients were evaluated with this scale (mean age: 21.5 years [6.58-43.42] and 21.29 years [6.42-42.25] respectively). Each item was videotaped and independently scored on a five-point scale by two pediatric neurologists (AC and VDP). In the case of a discrepancy between the two raters, the videos were watched again to come to an agreement. An average “oro-lingual score” and an average “gestural score” were determined. Given that a relatively large number of *ARX* patients came from two families (Family I and II), we also computed Family-weighted scores, by applying a weight to each *ARX* patient score for gestural and oro-lingual praxis, so that each family would contribute equally to the phenotyping. The weighted scores were thus less sensitive to the common genetic background.

#### Fine motor scales

Were administered to *ARX* and age-matched DS patients who participated in the kinematic study (Table 1). Family-weighted scores were also computed for the De Renzi scale, so that each family would contribute equally to the result.

**Table 1:** **Neuropsychological data in *ARX *** **patients and age-matched Down syndrome patients**

**Function evaluated**	**Test**	***ARX *** **patients**	**DS patients**	**Group comparison**
***Cognitive evaluation***				
	**Wechsler scale** (n = 16/16)^**1**^			
Intellectual quotient	Verbal IQ (VIQ)^2^	49 [45 – 61]	48 [45 – 60]	p = 0.33
	Performance IQ (PIQ)^2^	52 [45 – 60]	48 [41 – 56]	p = 0.28
	Total IQ (TIQ)	48 [40 – 70]	45 [40 – 54]	p = 0.28
**Raven’s coloured progressive matrices** (n = 16/16)				
Non-verbal reasoning	Mental age	6.9 years [4.2-11]	7.4 years [5.7-9.5]	p = 0.28
**Peabody Picture Vocabulary Test Revised (PPVT-R)** (n = 16/16)				
Receptive language	Vocabulary age	8.1 years	8.2 years	p = 0.96
***Adaptive and behavioural assessment***				
**Vineland adaptive behavioral scale** (n = 21/21)^**1**^				
Adaptive behavior	Global score	43 [20–74]	53 [33–72]	p = 0.02*
	Communication score	32 [20–64]	39 [21–65]	p = 0.07
	Daily-living skills	46 [20–108]	49 [20–82]	p = 0.38
	Socialization skills	49 [20–72]	70 [51–101]	p = 0.00002****
**Nisonger child behavior rating form**^**3**^ (n = 16/16)				
Number of patients using the 50^th^ percentile threshold				
Behavior disorder	Conduct disorder	5	0	p = 0.02*
	Anxiety	7	1	p = 0.018*
	Hyperactivity	5	0	p = 0.02*
	Automutilation/stereotyped behavior	6	2	NS
	Self-isolation/rituals	5	0	p = 0.02*
	Sensitivity/susceptibility	9	1	p = 0.003***
***Motor assessment***				
**Edinburgh handedness test** (n = 27/27)				
Handedness	Right- /Left-handed	19/7	26/1	p = 0.05*
**Videotaped protocol** (n = 21/21)				
Praxis skills	Orolingual praxis score	1.6	2.9	p = 0.00005****
	Gestural praxis score	2	3.1	p = 0.0003****
**De Renzi scale**^**4**^ (n = 12/12)				
Imitation of gestures	Global score	38 [14–56]	61 [54–70]	p = 0.00004****
	Number of patients scoring above the minimum normal score (>62)	0	5	p = 0.018*
	Number of patients scoring in the pathological range for apraxia (<53)	9	0	p = 0.0002***
	Fingers score	13 [2–24]	29 [22–35]	p = 0.00006****
	Limb score	25 [22–35]	32 [28–35]	p = 0.015*
**Lincoln-Oseretsky motor development scale**^**5**^ (n = 8/8)				
Psychomotor development	Global psychomotor developmental age	8.1 years [6–12]	7.4 years [6–9]	p = 0.45
	Manual precision	49 [25–75]	60 [38–75]	p = 0.18
	Global coordination	71 [29–100]	43 [14–71]	p = 0.028*
	Alternative movements	39 [0–100]	53 [0–100]	p = 0.42
	Speed of wrist/fingers movement	56 [29–100]	53 [29–86]	p = 0.83
	Balance	50 [25–75]	9 [0–50]	p = 0.003***
	Manual coordination	58 [0–100]	88 [67–100]	p = 0.05*

[Values] indicates the extreme values (minimum and maximum) obtained for each test.

For each scale, the normality of the data distribution was checked using the Shapiro-Wilk normality test. Then either an ANOVA or a non-parametric Mann and Whitney test was applied to compare differences across groups. The Fisher’s exact test was used to compare the number of left-handed patients and the number of patients with behavior disorder in ARX and DS groups.

*means <0.05, ***means <0.005 and ****means <0.001.

^**1**^Six out of 27 patients were excluded for the Vineland scale (3 younger than 5 y; 3 with severe epilepsy). Five others were excluded for the cognitive and behavioural assessment (3 Partington with severe dystonia; 2 refusals).

^**2**^Mean VIQ and PIQ were computed for patients who had WISC-III, WAIS-III, whereas only TIQ was computed for those who had a WISC-IV scale.

^**3**^[32,33].

^**4**^[34,35].

^**5**^[36-39].

#### Kinematic study

13 *ARX* patients and two control groups (healthy controls and DS patients) participated in the kinematic and brain MRI studies. The mean age of each group was 20.5 years [9.2-40.2], 21.30 [8.7-41.8] and 22.7 years [10.5-42.6] respectively.

**Figure 1:**
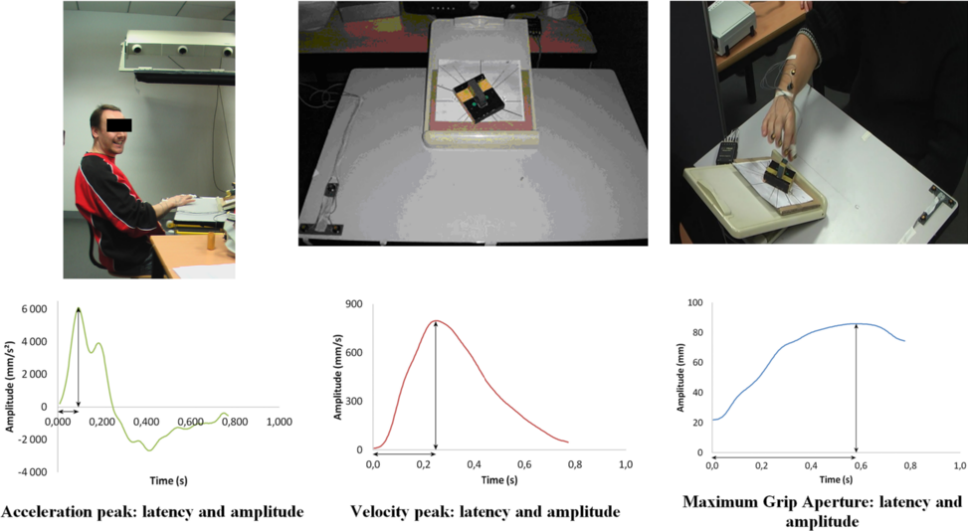
Kinematic task (set-up and kinematic parameters).

*Behavioral task:* The aim of this task was to reach, grasp and lift a plastic parallelepiped block (50*30*15 mm) from a specific starting point (Figure 1). Two variables were analyzed: the orientation of the object and the type of pinch. (i) The block could be set in two different orientations: +56° and −56°. (ii) Participants were instructed to successively grasp the object with three different pinches: thumb-index finger, thumb-middle finger, thumb-annular. For each condition, 10 movements were recorded, with a total of 60 movements.

*Movement recordings:* Six infrared light-emitting diodes (markers) were taped on the dominant hand: two on the wrist, one on the cubital part of the thumb nail, one on the radial part of the index finger, one on the middle finger, and one on the annular nail. Optotrak 3020 (Northern Digital) recorded the spatial position of the active markers at a frequency of 250 Hz with a spatial resolution of 0.1 mm. This task took 35 minutes to complete.

*Data analysis:* A second-order Butterworth dual pass filter (cutoff frequency, 10 Hz) was used for raw data processing. Individual movements were visualized and analyzed using Optodisp software [40]. A window of interest was defined as the time between the start of the movement and the time when the subject lifted up the block (see [41]) for detailed kinematic procedure). The following parameters were measured (Figure 1): movement duration (ms), latency (ms) and amplitude of the acceleration peak (mm/s^2^), velocity peak (mm/s), Maximum Grip Aperture (MGA: mm). Both transport and grasp components of movement were analyzed [42], by studying the wrist acceleration and the velocity peak for the transport component, and the MGA and opposition axis for the grasp component, respectively. For each parameter the average over the ten repetitions was calculated for each of the six conditions.

### Brain MRI

The patients were trained in a mock MRI scanner to decrease their anxiety and minimize movements during the scan. The acquisition was performed on a 1.5 T Siemens scanner (CERMEP, Lyon). High resolution (1×1×1 mm) structural imaging with a 3D T1-weighted Fast-Spoiled Gradient Recalled (FSPGR) sequence (TR 1970, TE 3.93 ms, TI 1100 ms, FOV 256*256) was obtained for each patient and age-matched healthy control. In addition, a T2 sequence (51 axial slices, FOV 220, voxel size: 0.9×0.9×2.5, TR 7740, TE 96) and a FLAIR sequence were acquired. The MRI session lasted 20 minutes. None of the patients required sedation during image acquisition. MRI images were clinically reviewed by two paediatric neurologists (AC and VDP).

### Statistical analysis

#### Clinical data

Statistical analysis was performed to compare the group of *ARX* patients with the age- and IQ-matched DS patients group in terms of gestural and oro-lingual praxis scores. As the data were ordinal, the non-parametric Mann and Whitney test was used. The Fisher’s exact test was used to compare the number of left-handed patients in *ARX* and DS groups. Results were considered significant at p < 0.05.

#### Neuropsychological battery and behavioral scales

For each scale, the normality of the data distribution was checked using the Shapiro-Wilk normality test. Then either an ANOVA or a non-parametric Mann and Whitney test was applied to compare differences across groups. Results were considered significant at p < 0.05.

#### Kinematic study

Kinematic parameters were analyzed within each group (*ARX*, DS, and Healthy Controls) using a repeated measure ANOVA with two within group factors: type of pinch and orientation. A between group analysis was also performed. A significance level of p < 0.05 was chosen. Post-hoc analysis was performed using a Newman-Keuls test. Correlations between kinematic parameters and De Renzi scale for upper-limb apraxia were analyzed using the Pearson test.

#### MRI study

The vermis height was determined on the T1-weighted sagittal medial view of each subject. The normality of the data distribution was checked using a Shapiro-Wilk normality test. An ANOVA was applied to compare differences across groups.

## Results

### Natural history and clinical features of the whole ARX group (27 patients)

*Pregnancy and neonatal history* were normal for all patients except three: one patient was born at 36 weeks of gestation, with macrosomy and neonatal hypoglycemia due to maternal diabetes mellitus, one had foetal bradycardia leading to an emergency cesarean section without neonatal neurological distress, and one had a materno-foetal infection with respiratory distress and early neonatal jaundice. The rate of cesarean section was 33%. Birth weight, height and head circumference were within the normal range.

*Developmental trajectory:* Unusual grasping was spontaneously reported by 82% of parents (e.g. “pinch with three fingers”, with a more important use of the middle finger and no use of the 4^th^ and 5^th^ fingers). All patients were able to walk, often with a mild delay (mean age of walking 20 months [min.12 – max.48]).

*Delayed language* was observed in all patients, but with a large variability. The mean age at which utterance of the first words occurred was 2.3 years [range 1–5 years], and the mean age at which they were able to combine two words was 3.9 years [range 2–8 years]. Among the 25 patients that were older than four years, 19 (76%) were able to produce intelligible sentences. 6 (24%) had very severe language impairment, either because of low cognitive level (3 patients (12%) with severe epilepsy, one of which had neonatal hypoglycemia and did not talk at all) or anarthria (3 patients (12%) with Partington syndrome). Drooling occurred during infancy in 56% of the patients and was still observed at the time of examination in 21%.

*Behavioral disorders* were noticed during early childhood in 17 of 27 patients (63%), including social withdrawal (41%), self aggressiveness (23%), hyperkinesia (17%), and temper tantrums (25%). Most of these symptoms disappeared during the second decade. Sleep problems were infrequent (15% multiple nightly awaking during early childhood). In teenage and adulthood, 22% were polyphagic, 11% had mood instability with depression, and 48% were considered as very anxious by the caregiver. However, only 7 out of 27 patients (26%) were on chronic psychotropic medications. Most of the patients were friendly, with a fine sense of humor. 9 out of 13 adult patients (69%) were working (8 (61%) in a sheltered workshop and 1 (8%) in an ordinary environment). Most of the adult patients could commute from home to work on their own.

*Epilepsy* was observed in 9 patients (33%). 3 patients (11%) presented with West syndrome (i.e. infantile spasms, psychomotor regression, and hypsarythmia on the EEG). Two of them, who had experienced a delay in the treatment of their infantile spasms, were severely mentally impaired and presented active epilepsy with multiple anticonvulsants (Additional file 3: Table S2). The third patient with West syndrome responded well to treatment (vigabatrin and corticosteroids) and his outcome did not differ from other dup24 patients without epilepsy. The remaining patients had various types of seizures (generalized tonic-clonic, tonic, complex partial seizures, and absences). Only 3 (33%) out of the 9 epileptic patients had sustained epileptic seizures requiring multiple antiepileptic drugs; 6 (67%) were seizure free (3 of them without treatment).

*Physical and sensory features:* Height, weight and head circumference growth were in the normal ranges. There was no obvious dysmorphia. However, striking common physical features were noticed (Additional file 4: Figure S2a and S2b) including high implantation of the hair or frontal baldness (even in very young patients), long face with thin saddle nose (with hypoplasic alae), and thin upper lip and mild retrognathism. Valgus feet in early childhood were common (70%). A deformation of the rachidian static with marked hyperlordosis and dorsal kyphosis was observed in 81% of teenagers and adults. Frequent ear infections occurred in 60%, which had lead to surgery in 31% (ear tube, adenoidectomy, amygdalectomy). No deafness was noticed. Refraction problems, mainly hyperopia, were found in 16% of cases without vision loss.

*Neurological examination and handedness:* In 46% of the 27 *ARX* patients, neurologic exams showed minor signs such as isolated Babinski sign or diffused tendon reflexes without any spasticity or motor deficit. Orolingual dyspraxia was mild in 18% of the patients and moderate to severe in 82%. Patients’ speech was often fast and jerky with a mild nasal intonation. Only 6 out of 24 patients older than 6 years (25%) were able to write and/or read short sentences. Ten (42%) could write their first name or a few words. Eight (33%) could neither write nor read. Calculation skills were generally weak, since only 7 out of 24 patients (29%) were able to perform addition and 4 patients (17%) were able to perform subtraction. Fine hand motor skills were impaired in all patients (mild in 40% of cases and moderate to severe in 60%). Hand grasping was very specific (see below). 19 of the 27 *ARX* patients (70%) were right-handed and 7 (26%) were strongly left-handed. One patient, who presented severe cognitive deficit, was ambidextrous. In comparison, only one of the 27 age-matched DS patients was left-handed and none of the 20 healthy boys from the *ARX* families was left-handed. The difference in the ratio of left- to right-handedness was statistically significant between *ARX* patients and healthy controls and also between *ARX* and DS patients (p = 0.014 and p = 0.05 respectively, Fisher exact test).

### Cognitive and behavioural profiles: comparison between ARX and DS patients

#### Cognitive phenotype

No statistically significant difference was found between the *ARX* and age-matched DS patients groups in measures of IQ (Wechsler scale), visual analogical reasoning mental age (Raven’s coloured Progressive Matrices test) and receptive language (PPVT-R) (Table 1).

#### Adaptive and behavioural profiles

The global adaptive score of the Vineland questionnaire was significantly higher in DS patients than in *ARX* patients (p = 0.022), mainly regarding social skills (Table 1).

The Nisonger child behaviour scales were analyzed using two different thresholds. When the 80^th^ percentile threshold was applied no significant group difference was observed. However, no DS patients but some ARX patients scored beyond the pathological threshold. When the 50^th^ percentile threshold was applied, the number of patients exhibiting conduct disorder, anxiety, hyperactivity, self-isolation/rituals, and sensitivity/susceptibility was significantly higher in the *ARX* group (Table 1).

### Motor assessment: comparison between ARX and DS patients

#### Videotaped oro-lingual and gestural praxis protocol in 21 ARX and 21 DS patients

All *ARX* patients older than 4 years and who could understand the task (n = 21, Table 1), as well as 21 age-matched DS patients, performed the praxis protocol.

**Figure 2:**
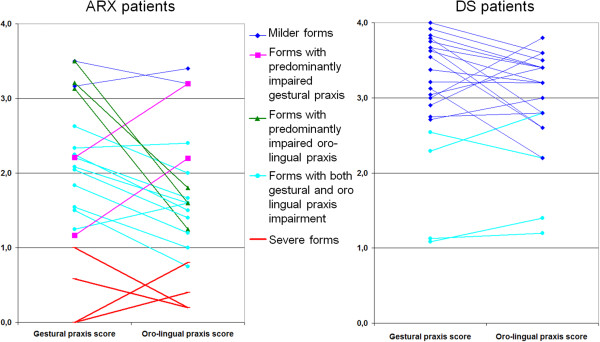
**Oro-lingual and gestural scores in 21 *ARX *** **mutated patients compared to 21 age-matched DS patients: 4 *ARX *** **patients (red lines) showed the most severe forms, with global scores below 1 for gestural and oro-lingual praxis; 15 *ARX *** **patients (blue, green and pink lines) had global scores between 1 and 2.5 with respect to oro-lingual and/or gestural praxis; 3 (green) had a predominantly gestural praxis impairment (difference between the two scores of more than 1), and 2 (pink) had a predominantly oro-lingual praxis impairment; the remaining 2 patients (dark blue) exhibited milder forms with scores just above 3.**

*Global scores in the ARX population: a continuum from mild to severe motor impairment*: All of the 21 *ARX* patients exhibited gestural and/or oro-lingual dyspraxia with a wide range of severity (Figure 2). No correlation was found between motor skills and age or cognitive level.

*Three striking “key features” of forearm and hand movements*: On average, scores for gestural praxis or oro-lingual praxis were significantly higher for DS patients than for *ARX* patients (Table 1). In the DS group, all except the two youngest patients (6:5 years and 7:7 years) scored above 2 (Figure 2). Family-weighted scores for gestural praxis and oro-lingual praxis were also significantly higher in DS patients than in *ARX* patients (Mann and Whitney, p = 0.0009 and p = 0.002 respectively).

**Figure 3:**
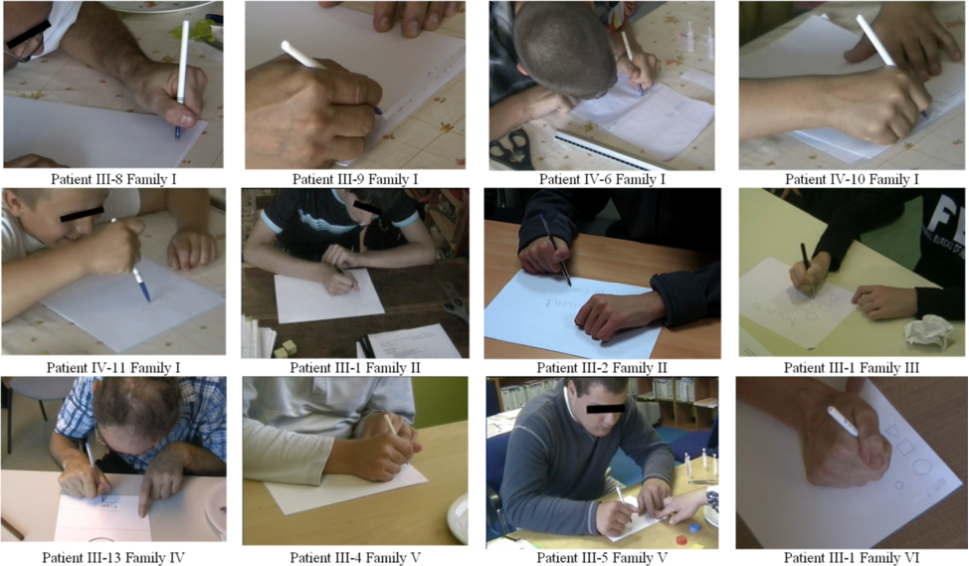
**Pen holding in *ARX *** **patients.**

(i) 20 out of the 21 *ARX* patients (95%) grasped the pen in a very peculiar way, including the severely affected patients with Partington syndrome for whom even grasping a felt-tip pen was very challenging, though they could easily kick a ball (Additional files 5 and 6). The typical “*ARX* pen holding” was between the lateral sides of the middle phalanx of index and middle fingers, and the thumb’s proximal phalanx (Figure 3). The pulps of thumb, index and middle finger were never involved in holding the pen. None of the DS patients presented this atypical pen holding. The only *ARX* patient who held the pen in contact with the pulp of the index had severe expressive language impairment with a mean oro-lingual praxis score of 1.3.

(ii) Rapid movements of pronation and supination of the wrist and forearm (i.e. alternating positioning of the palm and back of the hand; and moving one’s hands as glove puppets) were impaired in 15 (71%) out of 21 *ARX* patients (score < 2).

(iii) Use of the cubital part of the hand, specifically the fifth finger, was markedly impaired (i.e. opposition of the thumb and other fingers; grasping a bottle with the thumb and fifth finger).

#### Motor scales

**Figure 4:**
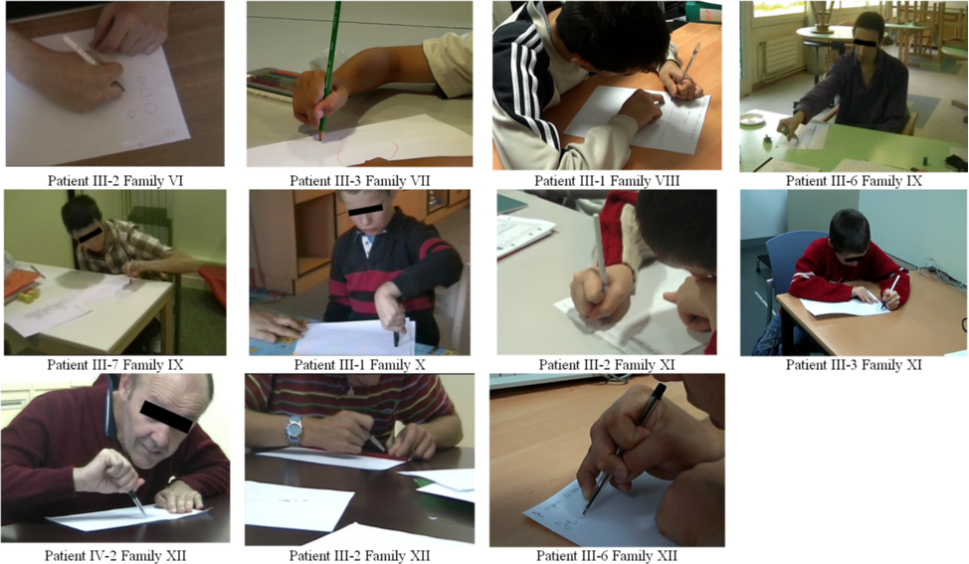
**Significant interaction between the factor ‘group’ and the within factor ‘effector’ (fingers vs limb) on the De Renzi scale: *ARX *** patients were much more impaired on independent fingers movements than on global limb movements (*p < 0.05; ****p < 0.0001).

A statistically significant group effect between *ARX* and DS patients was seen for the global score and all sub-tests of the De Renzi upper-limb apraxia scale with better scores for DS patients (Table 1). The ANOVA showed a significant effect of the factor ‘group’ (*ARX* vs. DS) but also an interaction between the factor ‘group’ and the within factor ‘effector’ (finger vs. limb), emphasizing a particular difficulty that *ARX* patients have with distal movements that require independent movement of the fingers, compared to limb movements (Figure 4, p = 0.016). The analysis of the type of error made for this test revealed that both groups of patients made sequential gesture errors, but *ARX* patients were significantly more clumsy (p < 0.05) and made more spatial errors (p < 0.001). Interestingly, *ARX* patients exhibited similar impairment when performing symbolic and non symbolic gestures, as well as when holding a position or executing motor sequences. Family-weighted ‘Global’, ‘Fingers’ and ‘Limb’ scores for the de Renzi scale were also significantly higher for DS patients than for *ARX* patients (Mann and Whitney, p = 0.007, p = 0.009 and p = 0.03 respectively).

The global psychomotor developmental age (Lincoln-Oseretsky Motor Development Scale) did not significantly differ between *ARX* and DS patients. However, DS patients had significantly lower scores than did *ARX* patients for ‘global coordination’ (which involves mainly the lower limbs) and ‘balance’ (Table 1). *ARX* patients had lower scores than did DS patients for hand coordination skills (Table 1).

#### Kinematic study

To further characterize *ARX* patients’ motor impairment, a kinematic study of a grasping movement was performed in 13 *ARX* patients, 13 age-matched healthy controls and 13 age-matched DS patients. We studied the effect of the orientation (+56° or −56°) and the type of pinch (thumb-index, thumb-middle finger and thumb-annular) on the movement duration and both the transport and the grasp components.

#### Group effect

Movement duration

A significant group effect was found [*F*(2,32) = 10.6, p < 0.001]: it took the *ARX* patients significantly more time to perform the movement than it took either the healthy age-matched controls (p < 0.001) or the DS patients (p < 0.01). Moreover, there was no statistically significant difference between DS and healthy age-matched controls on movement duration, despite the Intellectual Disability of DS patients.

Transport component

A significant group effect was found on wrist acceleration and velocity peaks latencies ([*F*(2,32) = 9.2, p < 0.001] and [*F*(2,32) = 9.8, p < 0.001] respectively). Post-hoc analysis revealed that both peaks occurred significantly later in *ARX* patients than in age-matched healthy controls and DS patients (Additional file 7: Table S3). The difference between DS patients and healthy controls was not significant for either of the two peaks.

Grasp component

A significant group effect was found on the Maximum Grip Aperture (MGA) latency [*F*(2,32) = 11.3, p < 0.001]. More precisely, post-hoc analysis showed that the maximum distance between grasping fingers occurred significantly later in *ARX* patients than age-matched healthy controls (p < 0.001), and age-matched DS patients (p < 0.005), while the difference between healthy controls and DS patients was not significant.

#### Orientation effect

Transport component

**Figure 5:**
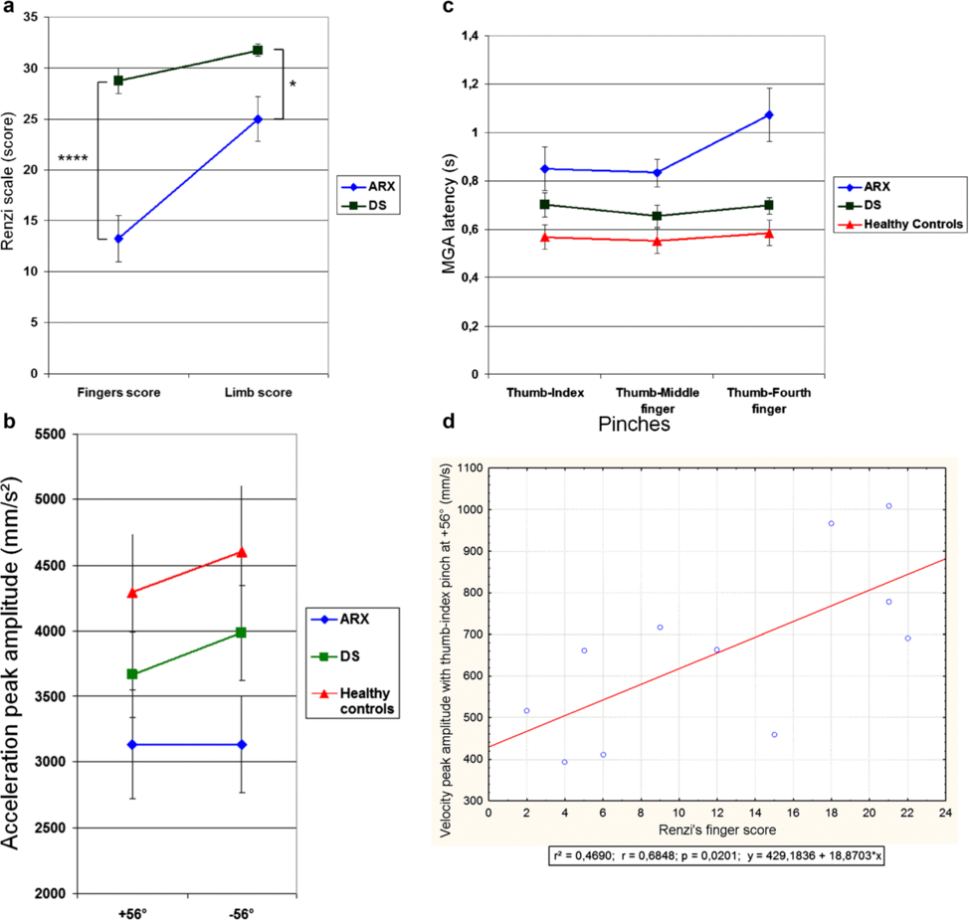
**Kinematic results. a**: Significant [object orientation*group] interaction on acceleration peak amplitude [*F*(2,32) = 3.45, p < 0.05]: a significant orientation effect was observed in healthy controls and DS patients but not in ARX patients; **b**: Significant [Pinch*group] interaction on MGA latency [*F*(4,64) = 7.06, p < 0.001]: ARX patients were impaired in using the thumb-fourth finger pinch; **c**: Significant positive correlation between the velocity peak amplitude with the thumb-index pinch at +56° and the De Renzi’s finger score.

A significant [object orientation*group] interaction was found on acceleration peak amplitude [*F*(2,32) = 3.5, p < 0.05]. In age-matched healthy controls and DS patients, a significant effect of object orientation was observed on both the acceleration peak amplitude and the velocity peak amplitude (Additional file 7: Table S3). When the object was oriented at −56°, both the acceleration peak and the velocity peak amplitudes were significantly greater than they were for the other orientation. By contrast, no orientation effect was observed in *ARX* patients on the acceleration peak amplitude or on the velocity peak amplitude (Figure 5a).

Grasp component

In age-matched healthy controls and DS patients, a significant effect of object orientation was observed on the MGA latency ([*F*(1,12) = 37, p < 0.001] and [*F*(1,12) = 9.9, p < 0.01] respectively) and amplitude ([*F*(1,12) = 106, p < 0.001] and [*F*(1,12) = 43, p < 0.001] respectively). In fact, when the object to grasp was oriented at −56°, the maximum distance between grasping fingers was significantly smaller and occurred significantly later than for the +56° orientation. By contrast, in *ARX* patients, no orientation effect was observed on the MGA latency or amplitude.

#### Type of pinch effect

Movement duration

A significant [pinch*group] interaction was found on movement duration [*F*(4,64) = 3.1, p = 0.02]: no pinch effect was observed in healthy controls and DS patients, whereas it was of borderline significance in *ARX* patients. *ARX* patients took significantly more time to perform the movement with the thumb-annular than with the thumb-middle finger pinch. Only 9 *ARX* patients out of the 13 who participated in the kinematic study were able to complete the task with the thumb-annular pinch whereas all the DS and Healthy age-matched controls completed it without difficulty (Fisher exact test, p < 0.05).

Transport component

The [type of pinch*group] interaction on velocity peak amplitude was statistically significant [*F*(4,64) = 2.8, p = 0.03]. Separate analysis revealed that a significant ‘type of pinch’ effect on velocity peak amplitude was observed for *ARX* patients but not for the other two populations (Additional file 7: Table S3). Post-hoc analysis further revealed that *ARX* patients had significantly greater velocity peak amplitudes with the thumb-middle finger pinch than with the thumb-index pinch (p = 0.04). This preference for the middle finger over the index was not observed in the other two groups.

Grasp component

The [pinch*group] interaction on MGA latency was significant [*F*(4,64) = 7.1, p < 0.001]. In both age-matched healthy controls and *ARX* patients, a significant effect of type of pinch was observed on MGA latency, but it was more pronounced in the *ARX* patients (Additional file 7: Table S3). When patients attempted to pick up the object with the thumb-annular pinch, the maximum distance between grasping fingers occurred significantly later in the movement than when patients attempted to pick up the object with the two other pinches (Figure 5b).

#### Correlations between De Renzi’s scores and kinematics data in ARX patients

Correlations between the kinematic parameters and De Renzi’s scale were analyzed in the 11 *ARX* patients who completed both tasks. A significant, linear positive correlation was found between the velocity peak amplitude with the thumb-index pinch and De Renzi’s finger score (Figure 5c). In fact, greater velocity peak amplitude of the thumb-index pinch correlated with better De Renzi’s finger score. A significant negative correlation was found between De Renzi’s finger score and the following kinematic parameters: velocity peak latency (R^2^ = 0.61, p = 0.02), MGA latency (R^2^ = 0.62, p = 0.02) and movement duration (R^2^ = 0.53, p = 0.04), when grasping with the thumb-annular pinch at −56°.

### Brain MRI study

13 *ARX* patients, 13 age-matched DS patients and 13 age-matched healthy controls completed a brain MRI. No major abnormalities were found in the *ARX* population. Non-specific MRI features were observed in 6 *ARX* and 5 DS patients (Additional file 8). Dilated Virchow Robin (VR) spaces were found in all *ARX* patients along the lenticulostriate arteries, and diffusively in one of them. It was not specific since 8 DS and 8 aged-matched healthy controls had also enlarged VR spaces.

All but one DS patient had vermis hypoplasia (Additional file 9: Figure S3). A significant group effect was found on the vermis height [*F*(2,36) = 56.249, p < 0.001] such that DS patients had significantly smaller vermis than the two other groups (Table 2). No significant difference in vermis height was found between *ARX* patients and healthy controls.

**Table 2:** MRI analysis

	**Healthy controls**	**ARX**	**DS**
**Mean vermis height (mm)**	47.2	47.8	37.3
**Standard deviation**	2.5	3.3	2.9
**Group effect**	[*F*(2,36) = 56.249, p < 0.001]****		
ARX/healthy controls	NS		
ARX/DS	p < 0.001****		
DS/healthy controls	p < 0.001****		

The normality of the data distribution was checked using a Shapiro-Wilk normality test. An ANOVA was applied to compare differences across groups.

****means <0.001.

## Discussion

### The Partington syndrome: a clinically recognizable disorder that includes all patients with a c.429_452dup24 of the *ARX gene*

The present study provides detailed clinical and neuropsychological data on 27 patients from 12 French families; all patients have been diagnosed with the same c.429_452dup24 mutation in the *ARX* gene between 2002 and 2006. The clinical features described in this series constitute a recognizable clinical syndrome specific to the c.429_452dup24 of *ARX*, which is useful for the diagnosis of unexplained mental retardation, even in sporadic cases. The present series is the first to describe the cognitive profile and specific motor impairments shared by all 27 *ARX* patients and demonstrates the existence of a pleiotropy. Patients with milder forms, who were erroneously labelled “non specific ID”, exhibit atypical handling and/or articulation impairment. Patients with the most severe form, already known as Partington syndrome, exhibit hand dystonia and/or anarthria [21]. In light of this spectrum of severity, we propose to consider the term of “Partington syndrome” for all patients affected with this mutation, to acknowledge M.W. Partington who first described the more severely impaired patients.

We report slightly lower epilepsy prevalence in our group (33%) compared to 45% in the Australian series [24]. The seizures exhibited by *ARX* patients in our study were of various types: tonic-clonic or complex partial seizures, mainly sensitive to current antiepileptic drugs. The frequency of infantile spasms (West syndrome) was 11% in our series, which is concordant with the 12.5% rate observed in the Australian series [24]. Response to corticosteroids appeared to be good if treated early, in contrast with infantile spasms associated with the c.333ins(GCG)_7_, which are usually drug resistant [14,15].

All *ARX* patients exhibited mild to severe orolingual dyspraxia. A fifth of *ARX* patients had very severe language impairment, either due to low cognitive level or to speech apraxia.

With regard to motor assessment, no true pyramidal syndrome was observed in *ARX* patients. This clinical sparing of the pyramidal tracts, contrasting with the major dystonic posture in some patients, is concordant with the expression pattern of *ARX*. Indeed, ARX is expressed in GABAergic neurons and is involved in tangential migration of GABAergic neurons from the telencephalon but not in radially migrating neurons, which gives rise to pyramidal neurons in the cortical plate.

As there is a positive correlation between the handedness of a child and the handedness of its biological parents [43], we chose to compare the *ARX* patients to the healthy controls (males) from their families. We reported a higher frequency of strong left-handedness in *ARX* patients compared to healthy controls and DS patients. In the literature, the great majority (90%) of the human population is right-handed [44]. This percentage contrasts with the one found in *ARX* patients (70%). The ontogenesis of handedness is usually thought as a multifactorial model [45]. ARX being a transcription factor expressed early in brain during the development, it could be interesting to test if ARX can regulate some of the 27 genes identified as asymmetrically expressed in the left and right hemisphere of 12-week-old human fetal brains [46].

The particular “reach and grip” impairment, which was observed in all *ARX* patients, was further characterized by the videotaped scale and kinematic data. Comparison of *ARX* patients and age-matched DS patients was useful in distinguishing between motor problems due to intellectual deficit and distortions linked to a specific *ARX* profile. For instance, both *ARX* and DS patients had lower acceleration and velocity peak amplitudes compared to age-matched healthy controls. This finding was most likely related to the Intellectual Disability of *ARX* and DS patients. However, all other parameters that were impaired in *ARX* patients were spared in DS patients.

Three key features affecting digits, hands, wrist and forearm movements are shared by *ARX* patients:

(i) Loss of preference of the index finger for grasping.

Ninety-five percent of the *ARX* patients in our series held a pen in a very specific way, between the lateral sides of the index and middle fingers, with the pen leaning upon the thumb’s proximal phalanx, without directly contacting the pulp of the fingers. Turner described this way of holding the pen as an “odd posture” of the hand in two unrelated families [30]. When they were presented with our kinematic task, *ARX* patients had significantly greater velocity peak amplitudes with the thumb-middle finger pinch than with the thumb-index finger pinch. This preference for the middle finger instead of the index was not observed in age-matched healthy controls and DS patients.

By studying the precision grasps of small beads of different sizes in 48 healthy children and 90 adults, Wong and Whishaw [47] described a high degree of variability in digit contact strategies, purchase patterns, and posture of the non grasping digits, depending on bead and hand size. However, a lateral grip missing any pulp contact of either the index or the thumb, such as observed in our *ARX* population, was never noticed. Similarly, among the various purchase strategies described, the use of the thumb and the middle finger, skipping the index, was very uncommon and considered improper. Conversely, the thumb-index pincer was used in more than 90% when grasping smaller beads, which were much more difficult to purchase [47].

(ii) Major impairment of fourth and fifth fingers deftness

It is interesting to note that the unusual grip posture spontaneously observed during the infancy of *ARX* patients by their parents (“pinch with three fingers” with a more important use of the major and no use of the 4^th^ and 5^th^ finger), persisted during adulthood, in an even more discrete way, as demonstrated in the kinematic study. In age-matched healthy controls and DS patients, the difficulty induced by the use of the thumb- fourth finger pinch, which is unusual, did not have any impact on the movement duration. By contrast, *ARX* patients took significantly more time to perform the movement with the thumb-fourth finger pinch than with the thumb-middle finger. Furthermore, the MGA latency for the thumb-fourth finger pinch was much more pronounced in *ARX* patients than in controls. In addition, only nine out of the thirteen *ARX* patients were able to complete the task with the thumb- fourth finger pinch though they completed the task utilizing the two other types of pinch without much trouble.

(iii) Lack of pronation/supination movements of the wrist and forearm.

In age-matched healthy controls and DS patients, but not in *ARX* patients, we found an orientation effect on both transport and grasp components. The +56° condition has been shown to be the easiest orientation for grasping an object [48]. At −56°, the movement duration was longer, the MGA occurred later and was smaller. This data suggests that the −56° condition was a more difficult orientation for grasping and required more fine motor control [49-51]. Acceleration and velocity peak amplitudes were higher at −56°. It is likely that the increase of acceleration and velocity peak amplitudes at −56° in healthy controls and DS patients compared to *ARX* patients is related to the pronation movement used by the DS and healthy controls to grasp the object at −56°. *ARX* patients did not show such an effect on either transport parameters or grasp parameters. In fact, *ARX* patients elevated their elbows rather than pronating their wrist in order to grasp the object at −56°. This observation is consistent with the finding that *ARX* patients exhibited impaired performances of rapid movements of pronation and supination of the wrist.

The term “focal dystonia” has often been used to describe the very specific hand grip of patients affected with Partington syndrome. However, the general definition of dystonia requires simultaneous co-contraction of agonist and antagonist muscles, leading to sustained hypertonia. In *ARX* patients, by contrast, weakness of muscles has been seen (but not stiffness) with a spontaneous position of the wrist at rest in a limp flexed position, both in the present and in past studies [24]. This awkward position at rest, which could have been mistaken for unusual hand mannerisms, does not fit with the definition of focal dystonia, which occurs during voluntary movement.

### Partington syndrome, a developmental model of Limb Kinetic Apraxia (LKA)

The term apraxia indicates an inability to perform purposeful movements in the absence of sensory-motor deficits or impaired understanding of what is required [52]. Classically, three types of limb apraxia have been identified: the ideational apraxia (patients do not know what to do), the ideomotor apraxia (patients know what to do but not how to do it), and Limb-Kinetic Apraxia (LKA) [52-54]. LKA was defined one century ago by Kleist, as “a loss of hand and finger dexterity resulting from inability to connect or isolate individual finger movements” [55]. The De Renzi upper-limb apraxia scale has been developed to distinguish LKA from ideomotor apraxia [35]. In our study, DS and *ARX* patients both exhibited a significant rate of ‘sequence’ errors, likely related to a common disability in motor programming resulting from their Intellectual Disability. Specifically, DS patients had significantly higher scores on the De Renzi scale than the *ARX* patients, and no DS patient scored below the pathological range for apraxia. Conversely, none of the *ARX* patients reached the minimum normal score, and 75% scored in the pathological range for apraxia. Furthermore, only *ARX* patients exhibited a high rate of awkwardness and spatial errors, similar to patients affected with LKA associated with CorticoBasal Degeneration (CBD) [35]. Interestingly, *ARX* patients fulfil two major clinical criteria for LKA diagnosis [54]: (i) impaired, coarse execution of simple movements of the hand, more evident distally than proximally and most notable for incoordination between fingers. It is striking to see how *ARX* patients were much more impaired in assessment of distal movement, which demanded independent finger movements, compared with more global movement, which involved the whole superior limb; (ii) impairment of all movements, i.e. symbolic/non symbolic, transitive/intransitive. *ARX* patients were similarly impaired in their performance of symbolic or non-symbolic gestures, and in holding a position or carrying out motor sequences. The core deficit in LKA is a distortion of individual finger movements and posture, similar to the peculiar *ARX* patients’ grasping behaviour. In addition, *ARX* patients exhibit buccofacial apraxia or at least tongue and lips movement impairment, as observed in patients with corticobasal degeneration [56,57].

The so called “LKA” observed in *ARX* subjects is a unique form of LKA for several reasons: *ARX*-related apraxia is (i) a “pure” LKA, without bradykinesia, rigidity and dystonia, three movement impairments commonly seen in corticobasal degeneration [54], allowing a better analysis of LKA pathophysiology; (ii) a bilateral LKA, by contrast with LKA in CBD which is mainly unilateral [54]; (iii) a developmental impairment observed as soon as in infancy, without obvious worsening with aging on retrospective data.

The pathophysiology of LKA suggests an impaired cortical inhibition for selection and control of hand muscular activity [53] and may be the result of loss of cortical inhibitory interneurons either in the frontal lobe or in the basal ganglia in CBD [53]. In Parkinson disease, the LKA seems also to be related to cortico-basal ganglia network dysfunction [58]. Since ARX is mainly expressed in inhibitory GABAergic interneurons of the developing cortex and striatum [59], a similar mechanism might be suggested in *ARX* mutated patients.

Visual inspection of the brain MRI of *ARX* patients did not reveal major basal ganglia abnormalities. The mild cystic images observed in most *ARX* patients located just beneath the putamen have the signal characteristics of CSF on MRI (Hyper T2, Hypo T1 and FLAIR images) and represent most probably a simple enlargement of the Virshow-Robin spaces along the lenticulo-striatal vessels. Nevertheless, it is questionable that obvious prominent cysts were already described in the posterior putamen of patients carrying the c.333ins(GCG)_7_ in the first polyalanine tract of the *ARX* gene [14]. Moreover, neuropathologic studies of the brain in newborn males affected with the XLAG syndrome (lack of ARX protein) show poorly delineated and atrophic basal ganglia and multiple small cavitations [5]. Besides, ARX has a major role in regulating basal ganglia differentiation in mice [60,61]. Interestingly, there was no difference in vermis height between *ARX* patients and healthy controls, while DS had a significantly smaller vermis compared to both groups. The normal cerebellar structure is concordant with the lack of expression of the *ARX* gene in the cerebellum [1] and good gross motor skills of *ARX* patients.

## Conclusion

The c.429_452dup24 mutation of the *ARX* gene constitutes a recognizable clinical syndrome: *ARX* patients exhibiting Intellectual Deficiency with no primary motor impairment, but with a very specific upper limb distal motor apraxia associated with a pathognomonic hand-grip. A continuum of severity level is observed ranging from the milder form, exhibiting atypical handling and/or articulation impairment, to the most severe form, with hand dystonia and/or anarthria. The *ARX* c.429_452dup24 mutation may be a developmental model for Limb Kinetic Apraxia.

## Competing interests

The authors declare that they have no competing interests.

## Authors’ contributions

AC contributed to the design of the study, examined clinically all the patients included in the study, contributed to the acquisition and analysis of the kinematic and MRI data, performed the statistical analysis of the neuropsychological, kinematic and MRI data and drafted the manuscript; TN contributed to the analysis of the kinematic data, the interpretation of the data and helped to draft the manuscript; AB contributed to the assessment and analysis of the cognitive, adaptive, behavioural and motor skills of the patients, contributed to the interpretation of the data and to the acquisition of the kinematic and MRI data; YP contributed to the design of the kinematic task, contributed to the acquisition of the kinematic and MRI data, contributed to the statistical analysis and to the interpretation of the kinematic data; AR contributed to the design of the study, to the interpretation of the data, and to draft the manuscript; KD contributed to the choice of the neuropsychological scales used in the study, contributed to the assessment and analysis of the cognitive, adaptive, behavioural and motor skills of the patients, contributed to the interpretation of the data, and contributed to the acquisition of the kinematic and MRI data; AC contributed to the acquisition of the MRI data, and contributed to the statistical analysis of the data; SB and FR contributed to the assessment and the analysis of the cognitive skills of the patients, and contributed to the acquisition of the MRI data; GB contributed to the choice of the neuropsychological scales used in the study, contributed to the assessment and analysis of the cognitive, adaptive, and behavioural skills of the patients, and contributed to the interpretation of the data; SM contributed to the clinical evaluation and interpretation of the data; DL, CC, BL, VL, ADSM, BGD, EB, DK, PB, FP, CRT and IM contributed to the clinical evaluation of *ARX* patients and interpretation of the data; MC and CP performed some of the molecular analysis and contributed to the interpretation of the data; SS contributed to the assessment and analysis of the cognitive, adaptive, and behavioural skills of some *ARX* patients; KP, TB and IS performed the molecular analysis and contributed to the interpretation of the data; BDF contributed to the design of the study, the clinical evaluation of DS patients, and interpretation of the data; MT contributed to the clinical evaluation of DS patients and interpretation of the data; IBC contributed to the design of the cognitive evaluation clinical evaluation of *ARX* patients and interpretation of the data; AT contributed to the clinical evaluation of *ARX* patients, interpretation of the data and draft of the manuscript; RT performed the molecular analysis, contributed to the clinical evaluation of DS patients and contributed to the interpretation of the data; DS contributed to the clinical evaluation of DS patients and contributed to the interpretation of the data; JC performed the molecular analysis, and contributed to the interpretation of the data; SF and JK contributed to the analysis of the data and were involved in the revision of the manuscript; NH contributed to the analysis, the interpretation of the data, and helped to draft the manuscript; RLG contributed to the analysis, the interpretation of the data, were involved in the revision of the manuscript; AR contributed to the design of the kinematic task, the acquisition and analysis of the kinematic data, contributed to the statistical analysis of the kinematic data, the interpretation of the data and the draft of the manuscript; VDP contributed to the design of the study, to the clinical evaluation of patients, to the interpretation of clinical, neuropsychological, kinematic and MRI data, and to the draft of the manuscript. All authors read and approved the final manuscript.

## Supplementary Material

Additional file 1: Figure S1 The genealogical tree of the twelve out of the 13 French c.429_452dup24 families diagnosed between 2002 and 2006, included in the study. Red arrow: patients who participated in the clinical study. Green asterisk: patients who were included in the clinical study but did not participate in the videotaped praxis study. Click here for file

Additional file 2: Table S1 Fifteen items videotaped Praxis scale. Click here for file

Additional file 3: Table S2 Epilepsy history of the 9 *ARX* patients with epilepsy. Click here for file

Additional file 4: Figure S2 Morphological features of *ARX* mutated patients: S2a: Faces of *ARX* patients clustered by families; S2b: Profiles of *ARX* patients clustered by families. Click here for file

Additional file 5 Video of an ARX patient easily kicking a ball. Click here for file

Additional file 6 Video of the same ARX patient showing his impairment in grasping a felt-tip pen. Click here for file

Additional file 7: Table S3 Kinematic results. Click here for file

Additional file 8 MRI data. Click here for file

Additional file 9: Figure S3 T1-weighted MRI sagittal view of the 13 Down syndrome patients: all but one had vermis hypoplasia. The green arrow shows the vermis height of a healthy control. Click here for file

## References

[B1] Bienvenu T Poirier K ARX, a novel Prd-class-homeobox gene highly expressed in the telencephalon, is mutated in X-linked mental retardation Hum Mol Genet 2002 11 8 981 991 11971879 10.1093/hmg/11.8.981

[B2] Stromme P Mangelsdorf ME Infantile spasms, dystonia, and other X-linked phenotypes caused by mutations in Aristaless related homeobox gene, ARX Brain Dev 2002 24 5 266 268 12142061 10.1016/s0387-7604(02)00079-7

[B3] Poirier K Lacombe D Screening of ARX in mental retardation families: consequences for the strategy of molecular diagnosis Neurogenetics 2006 7 1 39 46 16235064 10.1007/s10048-005-0014-0

[B4] de Brouwer AP Yntema HG Mutation frequencies of X-linked mental retardation genes in families from the EuroMRX consortium Hum Mutat 2007 28 2 207 208 17221867 10.1002/humu.9482

[B5] Bonneau D Toutain A X-linked lissencephaly with absent corpus callosum and ambiguous genitalia (XLAG): clinical, magnetic resonance imaging, and neuropathological findings Ann Neurol 2002 51 3 340 349 11891829 10.1002/ana.10119

[B6] Kitamura K Yanazawa M Mutation of ARX causes abnormal development of forebrain and testes in mice and X-linked lissencephaly with abnormal genitalia in humans Nat Genet 2002 32 3 359 369 12379852 10.1038/ng1009

[B7] Uyanik G Aigner L ARX mutations in X-linked lissencephaly with abnormal genitalia Neurology 2003 61 2 232 235 12874405 10.1212/01.wnl.0000079371.19562.ba

[B8] Kato M Das S Mutations of ARX are associated with striking pleiotropy and consistent genotype-phenotype correlation Hum Mutat 2004 23 2 147 159 14722918 10.1002/humu.10310

[B9] Bhat SS Rogers RC A novel in-frame deletion in ARX is associated with lissencephaly with absent corpus callosum and hypoplastic genitalia Am J Med Genet A 2005 138 1 70 72 16097002 10.1002/ajmg.a.30892

[B10] Guerrini R Marini C Genetic malformations of cortical development Exp Brain Res 2006 173 2 322 333 16724181 10.1007/s00221-006-0501-z

[B11] Kato M Saitoh S A longer polyalanine expansion mutation in the ARX gene causes early infantile epileptic encephalopathy with suppression-burst pattern (Ohtahara syndrome) Am J Hum Genet 2007 81 2 361 366 17668384 10.1086/518903 PMC1950814

[B12] Absoud M Parr JR A novel ARX phenotype: rapid neurodegeneration with Ohtahara syndrome and a dyskinetic movement disorder Dev Med Child Neurol 2009 52 3 305 307 19747203 10.1111/j.1469-8749.2009.03470.x

[B13] Wohlrab G Uyanik G Familial west syndrome and dystonia caused by an Aristaless related homeobox gene mutation Eur J Pediatr 2005 164 5 326 328 15726411 10.1007/s00431-005-1622-2

[B14] Guerrini R Moro F Expansion of the first PolyA tract of ARX causes infantile spasms and status dystonicus Neurology 2007 69 5 427 433 17664401 10.1212/01.wnl.0000266594.16202.c1

[B15] Poirier K Eisermann M Combination of infantile spasms, non-epileptic seizures and complex movement disorder: a new case of ARX-related epilepsy Epilepsy Res 2008 80 2–3 224 228 18468866 10.1016/j.eplepsyres.2008.03.019

[B16] Shinozaki Y Osawa M Expansion of the first polyalanine tract of the ARX gene in a boy presenting with generalized dystonia in the absence of infantile spasms Brain Dev 2009 31 6 469 472 18823727 10.1016/j.braindev.2008.08.006

[B17] Stromme P Mangelsdorf ME Mutations in the human ortholog of Aristaless cause X-linked mental retardation and epilepsy Nat Genet 2002 30 4 441 445 11889467 10.1038/ng862

[B18] Gecz J Cloosterman D ARX: a gene for all seasons Curr Opin Genet Dev 2006 16 3 308 316 16650978 10.1016/j.gde.2006.04.003

[B19] Shoubridge C Fullston T ARX spectrum disorders: making inroads into the molecular pathology Hum Mutat 2010 31 8 889 900 20506206 10.1002/humu.21288

[B20] Kato M Das S Polyalanine expansion of ARX associated with cryptogenic west syndrome Neurology 2003 61 2 267 276 12874418 10.1212/01.wnl.0000068012.69928.92

[B21] Partington MW Mulley JC X-linked mental retardation with dystonic movements of the hands Am J Med Genet 1988 30 1–2 251 262 3177452 10.1002/ajmg.1320300127

[B22] Frints SG Froyen G Re-evaluation of MRX36 family after discovery of an ARX gene mutation reveals mild neurological features of Partington syndrome Am J Med Genet 2002 112 4 427 428 12376949 10.1002/ajmg.10628

[B23] Gronskov K Hjalgrim H Screening of the ARX gene in 682 retarded males Eur J Hum Genet 2004 12 9 701 705 15199382 10.1038/sj.ejhg.5201222

[B24] Partington MW Turner G Three new families with X-linked mental retardation caused by the 428-451dup(24 bp) mutation in ARX Clin Genet 2004 66 1 39 45 15200506 10.1111/j.0009-9163.2004.00268.x

[B25] Stepp ML Cason AL XLMR in MRX families 29, 32, 33 and 38 results from the dup24 mutation in the ARX (Aristaless related homeobox) gene BMC Med Genet 2005 6 16 15850492 10.1186/1471-2350-6-16 PMC1142315

[B26] Gestinari-Duarte Rde S Santos-Reboucas CB ARX mutation c.428-451dup (24 bp) in a Brazilian family with X-linked mental retardation Eur J Med Genet 2006 49 3 269 275 16762829 10.1016/j.ejmg.2005.08.003

[B27] Fullston T Finnis M Screening and cell-based assessment of mutations in the Aristaless-related homeobox (ARX) gene Clin Genet 2011 80 6 510 522 21496008 10.1111/j.1399-0004.2011.01685.x

[B28] Abedini SS Kahrizi K Mutational screening of ARX gene in Iranian families with X-linked intellectual disability Arch Iran Med 2012 15 6 361 365 22642246

[B29] Arikan Y Bilgen T C.428_451 dup(24 bp) mutation of the ARX gene detected in a Turkish family Genet Couns 2012 23 3 367 373 23072184

[B30] Turner G Partington M Variable expression of mental retardation, autism, seizures, and dystonic hand movements in two families with an identical ARX gene mutation Am J Med Genet 2002 112 4 405 411 12376946 10.1002/ajmg.10714

[B31] Cossee M Faivre L ARX polyalanine expansions are highly implicated in familial cases of mental retardation with infantile epilepsy and/or hand dystonia Am J Med Genet A 2010 155A 1 98 105 21204215 10.1002/ajmg.a.33785

[B32] Aman MG Tasse MJ The Nisonger CBRF: a child behavior rating form for children with developmental disabilities Res Dev Disabil 1996 17 1 41 57 8750075 10.1016/0891-4222(95)00039-9

[B33] Tasse MJ Aman MG The Nisonger Child Behavior Rating form: age and gender effects and norms Res Dev Disabil 1996 17 1 59 75 8750076 10.1016/0891-4222(95)00037-2

[B34] De Renzi E Motti F Imitating gestures: a quantitative approach to ideomotor apraxia Arch Neurol 1980 37 6 10 7350907 10.1001/archneur.1980.00500500036003

[B35] Soliveri P Piacentini S Limb apraxia in corticobasal degeneration and progressive supranuclear palsy Neurology 2005 64 3 448 453 15699373 10.1212/01.WNL.0000150732.92567.BA

[B36] Sloan W The Lincoln-Oseretsky motor development scale Genet Psychol Monogr 1955 51 2 183 252 13251453

[B37] Vandenberg SG Factor analytic studies of the Lincoln-Oseretsky test of motor proficiency Percept Mot Skills 1964 19 23 41 14198854 10.2466/pms.1964.19.1.23

[B38] Bialer I Doll L A modified Lincoln-Oseretsky motor development scale: provisional standardization Percept Mot Skills 1974 38 2 599 614 4824100 10.2466/pms.1974.38.2.599

[B39] Rogé B Manuel de l'échelle de développement psychomoteur de Lincoln-Oseretsky 1984 Paris: Les Editions du Centre de Psychologie Appliquée

[B40] Thevenet M Paulignan Y “Optodisp.” 2001 copyright INSERM-CNRS-UCBL

[B41] Roy AC Paulignan Y Hand kinematics during reaching and grasping in the macaque monkey Behav Brain Res 2000 117 1–2 75 82 11099760 10.1016/s0166-4328(00)00284-9

[B42] Jeannerod M The timing of natural prehension movements J Mot Behav 1984 16 3 235 254 15151851 10.1080/00222895.1984.10735319

[B43] Reiss M Reiss G Earedness and handedness: distribution in a German sample with some family data Cortex 1999 35 3 403 412 10440077 10.1016/s0010-9452(08)70808-6

[B44] Corballis MC The evolution and genetics of cerebral asymmetry Philos Trans R Soc Lond B Biol Sci 2009 364 1519 867 879 19064358 10.1098/rstb.2008.0232 PMC2666079

[B45] Ocklenburg S Beste C Handedness: a neurogenetic shift of perspective Neurosci Biobehav Rev 2013 37 10 Pt 2 2788 2793 24091023 10.1016/j.neubiorev.2013.09.014

[B46] Sun T Walsh CA Molecular approaches to brain asymmetry and handedness Nat Rev Neurosci 2006 7 8 655 662 16858393 10.1038/nrn1930

[B47] Wong YJ Whishaw IQ Precision grasps of children and young and old adults: individual differences in digit contact strategy, purchase pattern, and digit posture Behav Brain Res 2004 154 1 113 123 15302117 10.1016/j.bbr.2004.01.028

[B48] Frak V Paulignan Y Orientation of the opposition axis in mentally simulated grasping Exp Brain Res 2001 136 1 120 127 11204406 10.1007/s002210000583

[B49] Chieffi S Gentilucci M Coordination between the transport and the grasp components during prehension movements Exp Brain Res 1993 94 3 471 477 8359261 10.1007/BF00230205

[B50] Bootsma RJ Marteniuk RG The speed-accuracy trade-off in manual prehension: effects of movement amplitude, object size and object width on kinematic characteristics Exp Brain Res 1994 98 3 535 541 8056073 10.1007/BF00233990

[B51] van Bergen E van Swieten LM The effect of orientation on prehension movement time Exp Brain Res 2007 178 2 180 193 17053908 10.1007/s00221-006-0722-1

[B52] Gross RG Grossman M Update on apraxia Curr Neurol Neurosci Rep 2008 8 6 490 496 18957186 10.1007/s11910-008-0078-y PMC2696397

[B53] Leiguarda RC Merello M Limb-kinetic apraxia in corticobasal degeneration: clinical and kinematic features Mov Disord 2003 18 1 49 59 12518300 10.1002/mds.10303

[B54] Zadikoff C Lang AE Apraxia in movement disorders Brain 2005 128 Pt 7 1480 1497 15930045 10.1093/brain/awh560

[B55] Kleist K Apraxie Jarbuch Psychiatr Neurol 1907 28 46 112

[B56] Lang AE Cortical basal ganglionic degeneration presenting with “progressive loss of speech output and orofacial dyspraxia” J Neurol Neurosurg Psychiatry 1992 55 11 1101 1469415 10.1136/jnnp.55.11.1101 PMC1015310

[B57] Ozsancak C Auzou P Dysarthria and orofacial apraxia in corticobasal degeneration Mov Disord 2000 15 5 905 910 11009198 10.1002/1531-8257(200009)15:5<905::aid-mds1022>3.0.co;2-d

[B58] Quencer K Okun MS Limb-kinetic apraxia in Parkinson disease Neurology 2007 68 2 150 151 17151340 10.1212/01.wnl.0000250331.35912.a5

[B59] Poirier K Van Esch H Neuroanatomical distribution of ARX in brain and its localisation in GABAergic neurons Brain Res Mol Brain Res 2004 122 1 35 46 14992814 10.1016/j.molbrainres.2003.11.021

[B60] Colombo E Collombat P Inactivation of Arx, the murine ortholog of the X-linked lissencephaly with ambiguous genitalia gene, leads to severe disorganization of the ventral telencephalon with impaired neuronal migration and differentiation J Neurosci 2007 27 17 4786 4798 17460091 10.1523/JNEUROSCI.0417-07.2007 PMC4916654

[B61] Friocourt G Parnavelas JG Mutations in ARX result in several defects involving GABAergic neurons Front Cell Neurosci 2010 4 4 20300201 10.3389/fncel.2010.00004 PMC2841486

