# Supplementary material for: The c.429_452 duplication of the ARX gene: a unique developmental-model of limb kinetic apraxia
Source: Orphanet J Rare Dis. 2014 Feb 14;9:25. doi: 10.1186/1750-1172-9-25 (PMC4016261; doi:10.1186/1750-1172-9-25)
Supplement: Additional file 1: Figure S1 — The genealogical tree of the twelve out of the 13 French c.429_452dup24 families diagnosed between 2002 and 2006, included in the study. Red arrow: patients who participated in the clinical study. Green asterisk: patients who were included in the clinical study but did not participate in the videotaped praxis study. [file 1750-1172-9-25-S1.pdf]

Supplementary Figure 1. Twelve out of 13 French 428_451dup (24pb) Families diagnosed between 2002 and 2006, included in the study. Red arrow: patients who participated to the clinical study. Green asterisk: patients who were included in the clinical study but did not participate to the videotaped praxis study.

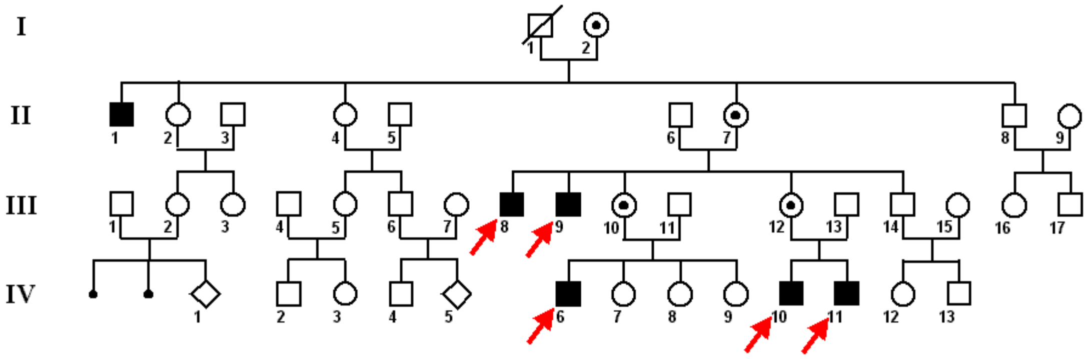

Family I. Five of 6 patients available;
published as “P73, non specific MR” in Bienvenu et al., 2002)

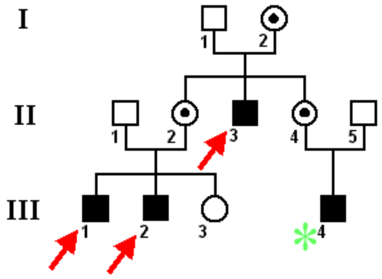

Family II. Four patients available, one too young for videotape study. (published as P49, “MR with mild hand dystonia” in Bienvenu et al., 2002)

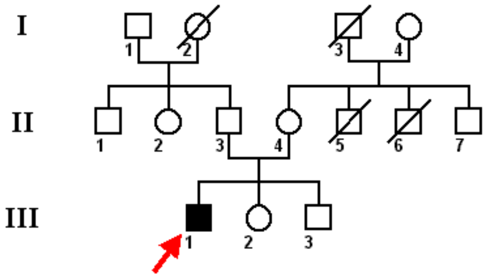

Family III. One sporadic case with speech apraxia and Partington syndrome (unpublished)

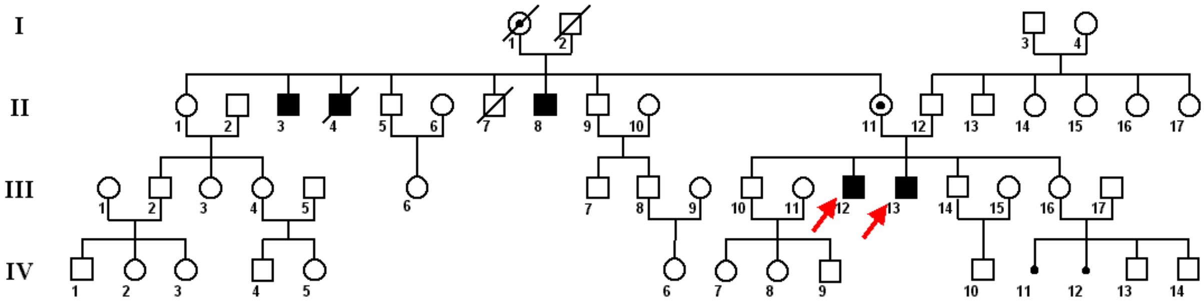

Family IV. Two of 4 alive patients available, the other two were too old
(published as P104, “non specific MR” in Poirier et al., 2006)

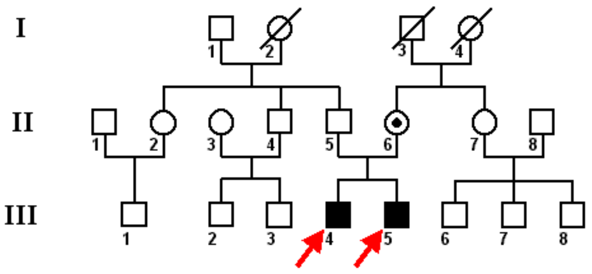

Family V. A brother pair, one too severe for videotape study, unpublished.

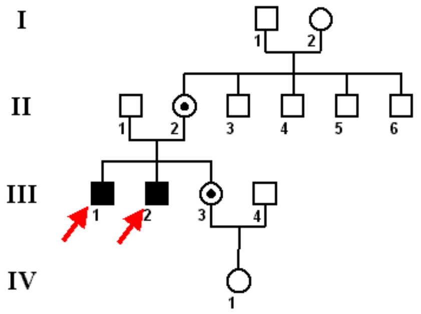

Family VI. Published as P106, “non specific MR”, in Poirier et al., 2006.

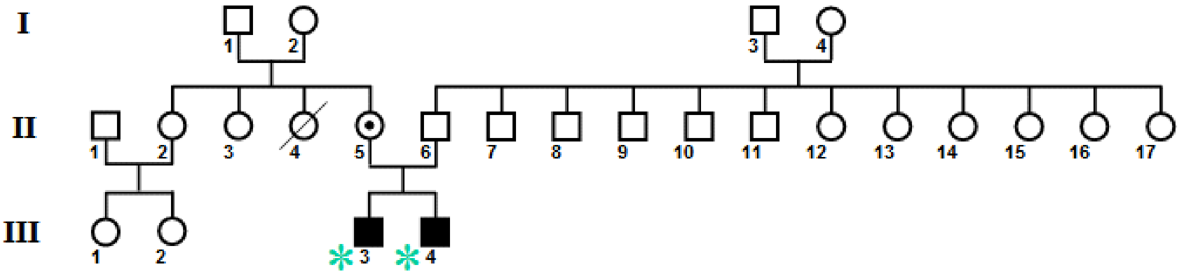

Family VII. Two boys available, both too young for videotape study; unpublished.

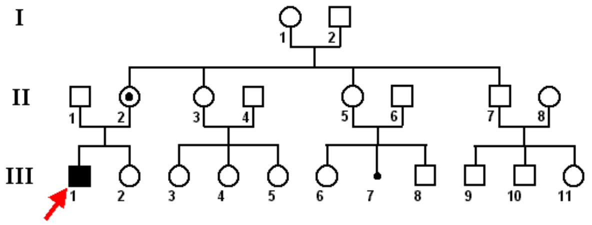

Family VIII. Sporadic case, mild hand dyspraxia; unpublished.

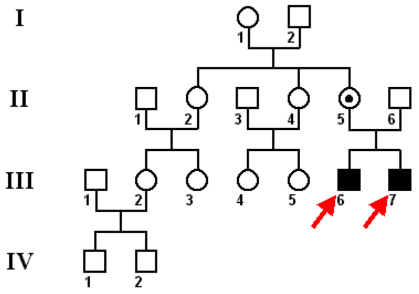

Family IX. Partington, P34 in Poirier et al., 2006)

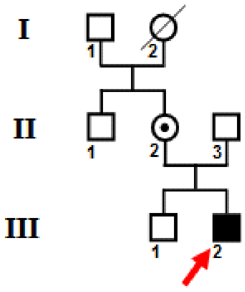

Family X. One sporadic case too severe for videotape study, in Cossee et al, 2010.

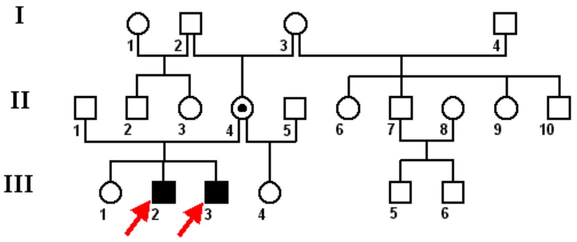

Family XI. Two available patients, unpublished

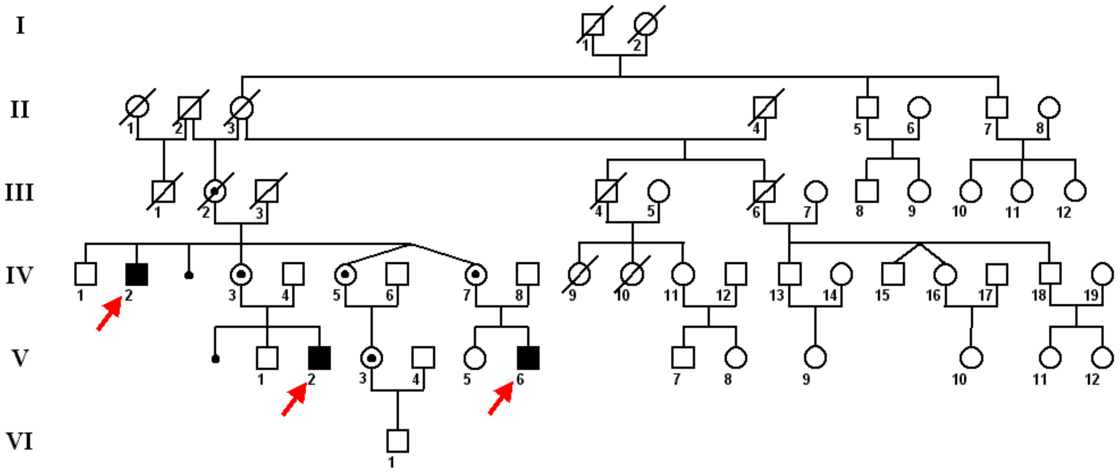

Family XII. Three patients available, one too severely affected did not participate to the videotape study; published as T37 « Non specific MR » in Poirier et al., 2006.
