# Supplementary material for: The c.429_452 duplication of the ARX gene: a unique developmental-model of limb kinetic apraxia
Source: Orphanet J Rare Dis. 2014 Feb 14;9:25. doi: 10.1186/1750-1172-9-25 (PMC4016261; doi:10.1186/1750-1172-9-25)
Supplement: Additional file 2: Table S1 — Fifteen items videotaped Praxis scale. [file 1750-1172-9-25-S2.pdf]

Supplementary Table 1. Fifteen items videotaped Praxis scale.

**Gestural score**	**Oro-lingual score**
1. Holding a pen<br>2. Piling up 8 blocks<br>3. Wrist and forearm pronation / supination<br>4. Moving hand (glove puppets)<br>5. Buttoning up coat<br>6. Tying shoe-laces<br>7. Sequence of three hand gestures (palm, edge, fist)<br>8. Cutting a sheet of paper (grasping, cutting and releasing a pair of scissors)<br>9. Opposition of the thumb and other fingers (2^d to 5th)<br>10. Grasping a small bottle with the thumb and 5th finger	1. Producing a kiss<br>2. Blowing out cheeks<br>3. Moving the tongue up, down, left and right<br>4. Picture naming (quality of pronunciation)<br>5. Sentence repetition (number of recognizable syllabes)

Suppl MRI Data (or data not schown):

Two ARX patients had a mild ventriculomegaly (lateral ventricule were 11 and 12mm wide respectively), two had ventricular asymmetry without dilatation, one had postero-superior vermis atrophy (lobules VI and VII) associated with an enlargement of the horizontal hemispherical cerebellar fissure and one ARX patient had a retrocerebellar arachnoidal cyst.

Two DS patients had cavum septum lucidum and cavum vergae, one had mega cisterna magna, one had small ventricular dilatation (lateral ventricule was 11mm wide), and one had ventricular asymmetry without dilatation.

In addition, one healthy control had an enlargement of the horizontal hemispherical cerebellar fissure.
