# Supplementary material for: The c.429_452 duplication of the ARX gene: a unique developmental-model of limb kinetic apraxia
Source: Orphanet J Rare Dis. 2014 Feb 14;9:25. doi: 10.1186/1750-1172-9-25 (PMC4016261; doi:10.1186/1750-1172-9-25)
Supplement: Additional file 3: Table S2 — Epilepsy history of the 9 ARX patients with epilepsy. [file 1750-1172-9-25-S3.pdf]

Table 2. Epilepsy history of the 9 ARX patients with epilepsy.

Patient	Type of seizure	Age at onset	Frequency and circumstances of seizures	EEG	medications	Outcome of epilepsy	Severity of cognitive deficit
1<br>(Family I)	**Infantile spasms**	4 months	daily	hypsarythmia	GVG, HC<br>Topiramate	At 20 months: free of seizure and meds	Moderate
2<br>(Family III)	Partial complex	4 years and 4 months	Total of 8 seizures<br>(1 watching TV)	Occipitotemporal spikes & slow waves, generalized spike wave discharges	VPA	At 17 years and 9 months: free of seizures on VPA	Moderate
3<br>(Family V)	- **Infantile spasms**<br>- Febrile status epilepticus<br>- generalized tonic clonic seizure<br>- partial seizure	- 5 months<br>- 14 years and 9 months<br>- 17 years and 3 months<br>- 17 years and 7 months	- daily<br>- Rare generalized tonic clonic seizures	hypsarythmia	ACTH, CZP<br>VPA	At 18 years and 8 months: last seizure at 17 years 7 months old, on VPA CZP	**profound**
4<br>(Family VII)	Short febrile tonic clonic<br>(once without fever)	3 years and 4 months	Total of 3 seizures within six months	normal	VPA	At 4 years and 6 months: free of seizures on VPA	Moderate
5<br>(Family X)	- **Infantile spasms**<br>- partial complex seizures<br>- noctural tonic-clonic<br>- generalized tonic-clonic seizures<br>- status epilepticus	- 7 months<br>- 22 months<br>- 3 years and 2 months<br>- 5 years and 8 months<br>- 6 years and 1 month	- Daily<br>- Twice a week<br>- Once a week<br>- every two months	Na	- HC ACTH<br>- CBZ<br>- GVG<br>- VPA<br>- LTG LVT	At 11 years and 4 months: generalized tonic clonic seizure once a month, on VPA, LTG, LVT	**profound**
6<br>(Family XI)	Absences	4 years	Some watching TV	Na	VPA	At 11 years and 10 months: free of seizures and meds	Moderate
7<br>(Family XI)	- Febrile seizure<br>- Absences	- 14 months<br>- 7 years and 9 months	watching TV	Na	no	At 7 years and 11 months: free of seizures and meds	Moderate
8<br>(Family XII)	**?**	14 years			Ethosuccimide<br>VPA, CLB	At 54 years: occasionally	severe

9<br>(Family XII)	- Hypotonic seizures<br>- Tonic seizures with eye rolling, pallor and cyanosis	- 22 months<br><br>- 5 years	- once<br><br>- 2 to 3 times a week	Normal<br>at 4 years old	- VPA CBZ CLB	At 18 years and 6 months: free of seizures on VPA CBZ	severe

CBZ: carbamazepin; CZP: clonazepam; CLB: clobazam; LTG: lamotrigin; VPA: valproate; GVG: vigabatrin; LVT: levetiracetam; HC: hydrocortisone.
