# Supplementary figures and images for: The c.429_452 duplication of the ARX gene: a unique developmental-model of limb kinetic apraxia
Source: Orphanet J Rare Dis. 2014 Feb 14;9:25. doi: 10.1186/1750-1172-9-25 (PMC4016261; doi:10.1186/1750-1172-9-25)

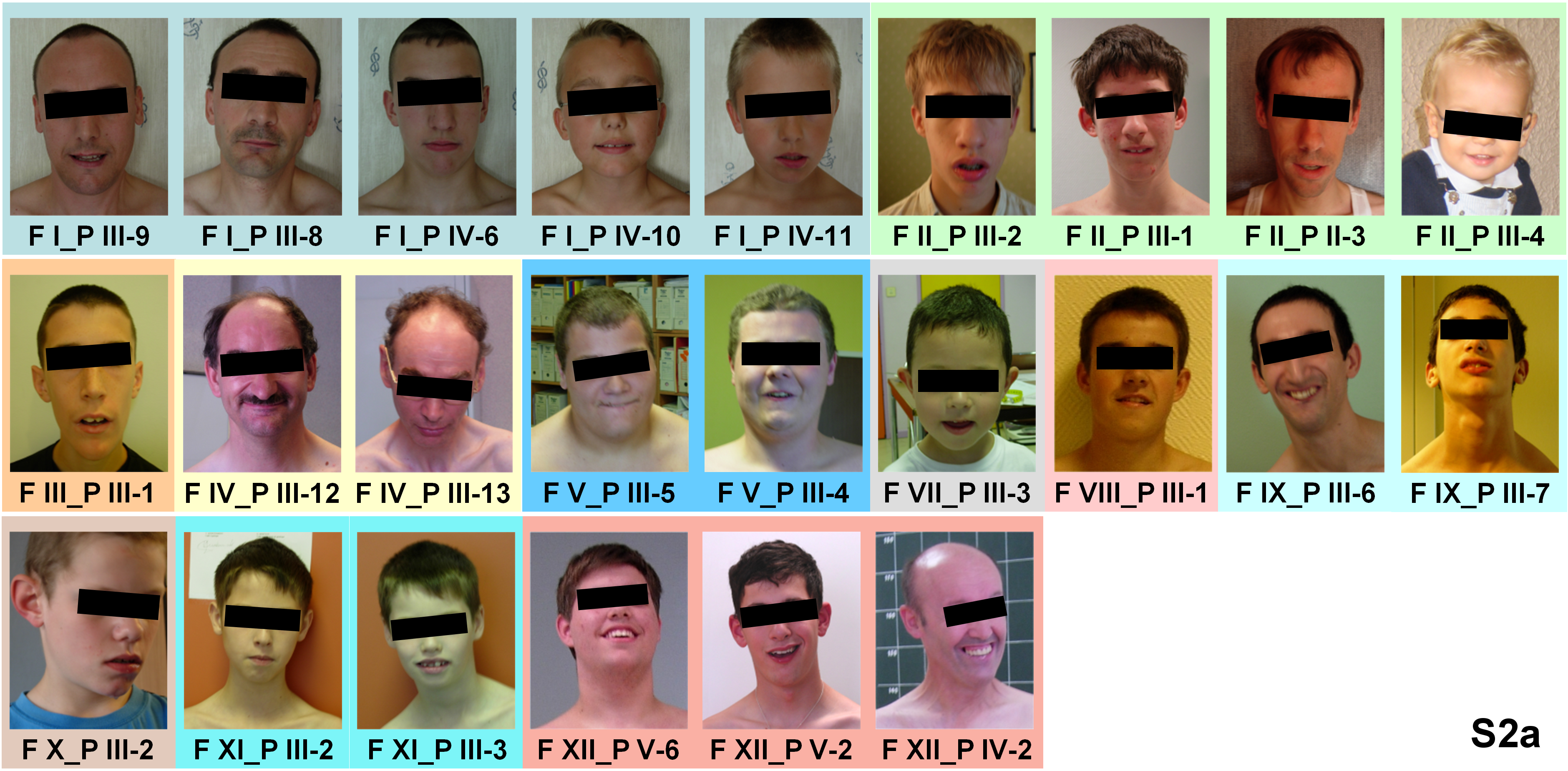

Supplement: Additional file 4: Figure S2 — Morphological features of ARX mutated patients: S2a: Faces of ARX patients clustered by families; S2b: Profiles of ARX patients clustered by families. [file 1750-1172-9-25-S4.tiff]

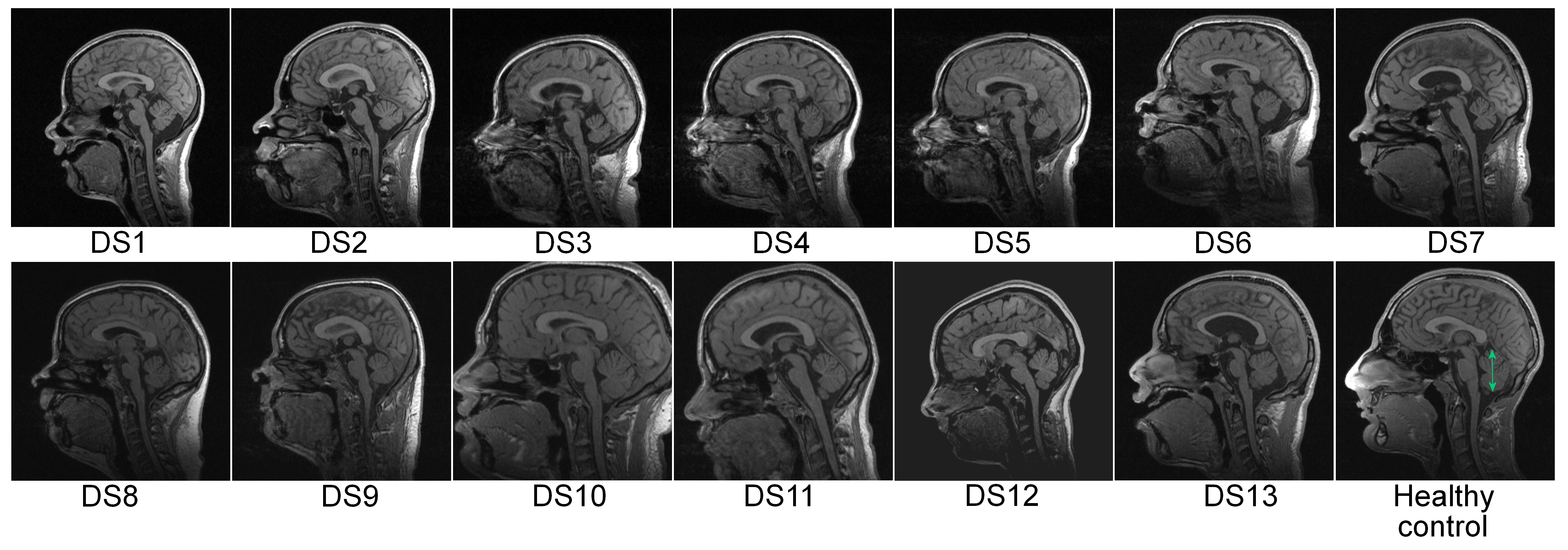

Supplement: Additional file 8 — MRI data. [file 1750-1172-9-25-S8.tiff]

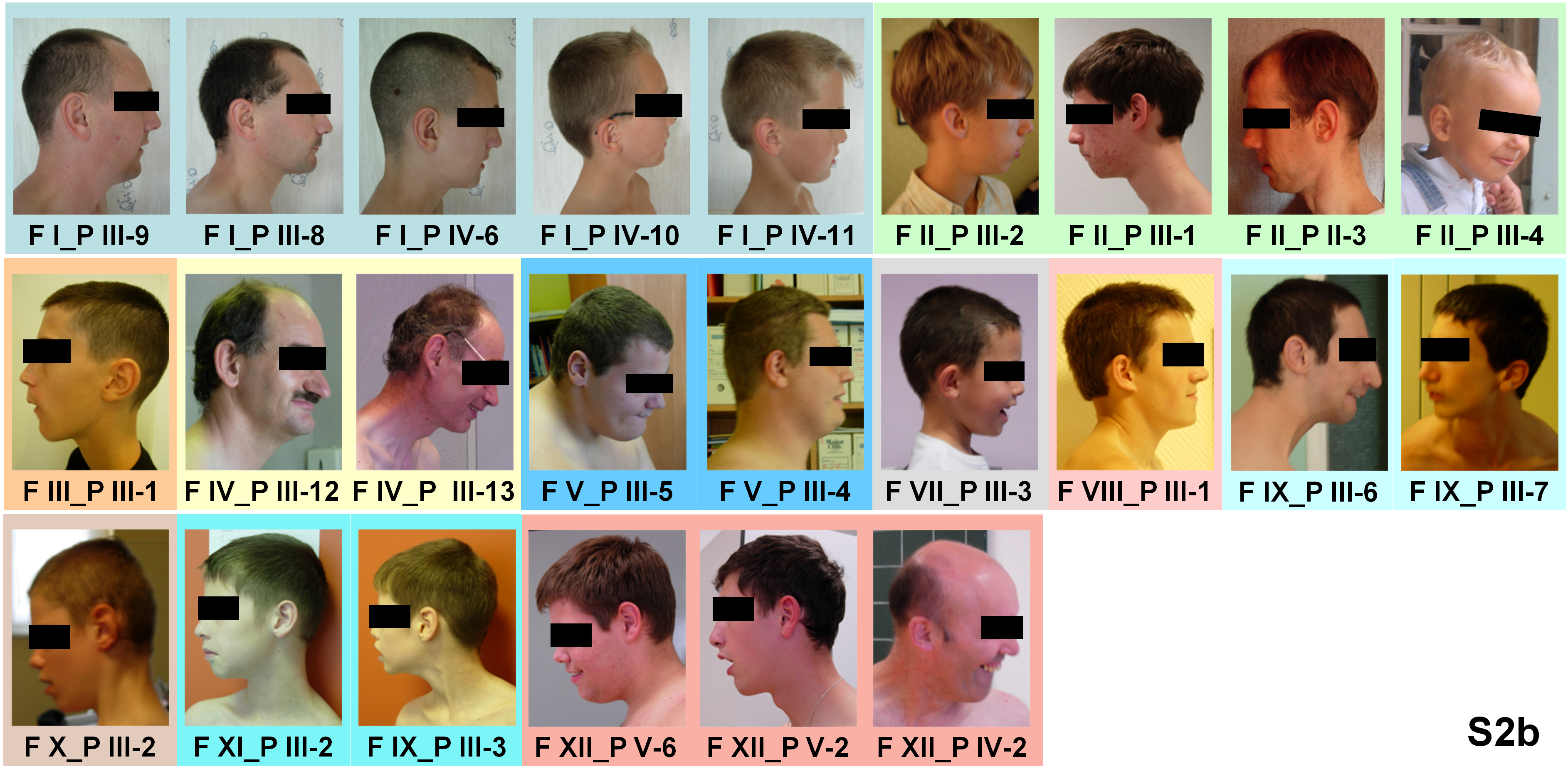

Supplement: Additional file 9: Figure S3 — T1-weighted MRI sagittal view of the 13 Down syndrome patients: all but one had vermis hypoplasia. The green arrow shows the vermis height of a healthy control. [file 1750-1172-9-25-S9.tiff]
