# Supplementary material for: The c.429_452 duplication of the ARX gene: a unique developmental-model of limb kinetic apraxia
Source: Orphanet J Rare Dis. 2014 Feb 14;9:25. doi: 10.1186/1750-1172-9-25 (PMC4016261; doi:10.1186/1750-1172-9-25)
Supplement: Additional file 7: Table S3 — Kinematic results. [file 1750-1172-9-25-S7.pdf]

Table 3: Kinematic results

	**Movement duration (ms)**	**Transport component**				**Grasp component**	
		**Acceleration peak**		**Velocity peak**		**MGA**	
		**Latency (ms)**	**Amplitude (mm/s^2)**	**Latency (ms)**	**Amplitude (mm/s^2)**	**Latency (ms)**	**Amplitude (mm)**
**Healthy controls**	800	220	4668	341	853	568	90.37
**DS**	988	259	3180	383	701	683	89.09
**ARX**	1284	326	2785	473	670	978	89.98
**Group effect**	[F(2,32)=10.6, p<0.001]****	[F(2,32)=9.2, p<0.001]****	[F(2,32)=6, p<0.01]**	[F(2,32)=9.8, p<0.001]****	[F(2,32)=3.5, p<0.05]*	[F(2,32)=11.3, p<0.001]****	NS
ARX/healthy controls	p<0.001****	p<0.001****	p<0.01**	p<0.001****	p<0.05*	p<0.001****	
ARX/DS	p<0.01**	p<0.01**	NS	p<0.01**	NS	p<0.005***	
DS/healthy controls	NS	NS	p<0.05*	NS	p<0.05*	NS	
**Orientation effect**	[F(1,32)=31.3, p<0.0001]****	NS	[F(1,32)=4, p<0.05]*	NS	[F(1,32)=16, p<0.0005]****	[F(1,32)=17.2, p<0.0005]****	[F(1,32)=45.6, p<0.0001]****
**[Orientation*group] interaction**	NS	NS	[F(2,32)=3.5, p<0.05]*	NS	NS	NS	NS
Healthy controls	[F(1,12)=20.7, p<0.001]****	NS	[F(1,12)=7, p<0.05]*	NS	[F(1,12)=18.8, p<0.001]****	[F(1,12)=37, p<0.001]****	[F(1,12)=106, p<0.001]****
DS	[F(1,12)=32.3, p<0.0001]****	NS	[F(1,12)=6.3, p<0.05]*	NS	[F(1,12)=7, p<0.05]*	[F(1,12)=9.9, p<0.01]**	[F(1,12)=43, p<0.001] ****
ARX	[F(1,8)=5.3, p<0.05]*	NS	NS	NS	NS	NS	NS
**Type of pinch effect**	[F(2,64)=8.1, p<0.001]****	NS	NS	NS	NS	[F(2,64)=14.9, p<0.0001]****	[F(2,64)=7.8, p<0.001]****
**[Type of pinch*group] interaction**	[F(4,64)=3.1, p=0.02]*	NS	NS	NS	[F(4,64)=2.8, p=0.03]*	[F(4,64)=7.1, p<0.001]****	NS
Healthy controls	NS	NS	NS	NS	NS	[F(2,24)=4.4, p<0.05]*	[F(2,24)=9.23, p<0.005]***
DS	NS	NS	NS	NS	NS	NS	F(2,24)=3.01, p<0.06]
ARX	[F(2,16)=3.3, p=0.06]	NS	NS	NS	[F(2,16)=3.6, p=0.049]*	[F(2,16)=9.4, p<0.005]***	NS

NS: Non Significant; * means <0.05, ** means <0.01, *** means <0.005 and **** means <0.001

Kinematic parameters were analyzed within each group (ARX, DS, and Healthy Controls) using a repeated measure ANOVA with two within group factors: type of pinch and orientation. A between group analysis was also performed. Post-hoc analysis was performed using a Newman-Keuls test.
